# New Levels of Language Processing Complexity and Organization Revealed by Granger Causation

**DOI:** 10.3389/fpsyg.2012.00506

**Published:** 2012-11-19

**Authors:** David W. Gow, David N. Caplan

**Affiliations:** ^1^Neuropsychology Laboratory, Massachusetts General HospitalBoston, MA, USA; ^2^Department of Psychology, Salem State UniversitySalem, MA, USA; ^3^Athinoula A. Martinos Center for Biomedical Imaging, Massachusetts General HospitalCharlestown, MA, USA; ^4^Harvard-Massachusetts Institute of Technology Division of Health Sciences and TechnologyCambridge, MA, USA

**Keywords:** Granger causation analysis, MEG, EEG, effective connectivity, rich-club connectivity, small world connectivity, superior temporal gyrus, speech perception

## Abstract

Granger causation analysis of high spatiotemporal resolution reconstructions of brain activation offers a new window on the dynamic interactions between brain areas that support language processing. Premised on the observation that causes both precede and uniquely predict their effects, this approach provides an intuitive, model-free means of identifying directed causal interactions in the brain. It requires the analysis of all non-redundant potentially interacting signals, and has shown that even “early” processes such as speech perception involve interactions of many areas in a strikingly large network that extends well beyond traditional left hemisphere perisylvian cortex that play out over hundreds of milliseconds. In this paper we describe this technique and review several general findings that reframe the way we think about language processing and brain function in general. These include the extent and complexity of language processing networks, the central role of interactive processing dynamics, the role of processing hubs where the input from many distinct brain regions are integrated, and the degree to which task requirements and stimulus properties influence processing dynamics and inform our understanding of “language-specific” localized processes.

## Introduction

Experimental methods inherently shape the way we understand the world and the kinds of questions we ask as researchers. To early aphasiologists including Wernicke (1874/1969), observations of the deficits associated with focal brain damage combined with inferences based on apparent functional dependencies between different brain regions led to the view that language is the product of a dynamic, distributed network of specialized processors. A century later, developments in neuroimaging have shaped a neuroscience that until recently has focused primarily on the interpretation of local function rather than dynamic interactions between localized processors. This focus reflects classical BOLD imaging techniques that reliably, and with increasing precision, isolate and localize functions, but are inherently limited in their ability to identify the direction of processing interactions or to distinguish between function-specific and neuromodulatory effects (Logothetis, 2008). This trend has shifted in recent years with the development of new techniques for identifying patterns of directed causal interaction within networks, or effective connectivity, using a variety of imaging modalities (Friston et al., 1997, 2003; Bullmore, 2000).

In our work (Gow and Segawa, 2007, 2009; Gow et al., 2008; Caplan and Gow, 2012; Gow and Nied, 2012), we have applied a specific effective connectivity analysis, Granger analysis of MRI-constrained magnetoencephalography (MEG)/electroencephalography (EEG) data, to the study of interactions that support the perception of speech and sentence processing. This paper is intended as an introduction to these techniques and a discussion of the implications that our results and, more broadly, the nature of these analyses themselves hold for the way we understand and approach the study of language processing.

## Functional Architecture, Effective connectivity, and Causal inference

Measures of functional connectivity identify correlated or coordinated activity. In contrast, measures of effective connectivity identify causal, directed influences between brain regions. They are used to determine how (and ideally when) activity in one brain region drives activity in another. When coupled with well-developed analysis of localized function, effective connectivity analyses offer a direct view of the functional architecture of cognitive processes. They can be used to resolve fundamental questions including whether processing is modular or interactive, parallel or serial, or organized into streams or hubs.

Significantly, functional architecture are not readily addressed by either behavioral or neuroimaging techniques. In behavioral paradigms subjects are generally presented with a stimulus and the dependent measure is the latency or accuracy of a judgment. Unfortunately, there is no way to know whether the judgment reflects the state of one particular representation (e.g., acoustic-phonetic or lexical) or the output of a response selection mechanism that may be informed by the state of one or perhaps several of these representations (Dennett, 1992; Norris et al., 2000). Moreover, with the exception of eyetracking and mousetracking paradigms, behavioral paradigms typically only examine the output of processing and so provide no direct window on the dynamics that lead up to the judgment. The problem is further exacerbated by the potential for a tradeoff between assumptions about structure and processing (Anderson, 1978) that limits the strength of inferences about functional architecture based on purely chronometric data.

Traditional neuroimaging data are similarly limited. Consider a hypothetical result in which BOLD imaging reveals correlated activation between brain regions associated with acoustic-phonetic processing and lexical representation. Such a result could be produced by bottom-up activation of lexical areas by acoustic-phonetic areas. It could also reflect activation of acoustic-phonetic areas by lexical areas, a reciprocal interaction between both areas, or spurious correlation with no underlying causal link between the regions. Electrophysiological data offer a variant of the same problem. If activation of the acoustic-phonetic region occurs before activation of the lexical area it is tempting to infer that the later activation was caused by the earlier activation. However, the same temporal patterning could result from a difference in the rise times of lexical and acoustic-phonetic processing. It could also be a purely accidental property. As a result, inferences about functional architecture depend on the formal apparatus of effective connectivity analysis.

Analyses of effective connectivity are rooted in intuitions about causality. It is important to remember that statistics alone cannot directly determine whether one variable causes changes in another. At best, they can be used to identify and quantify patterns that are the basis of human attributions about causality. One way we discover causality is through systematic experimentation. We can systematically perturb a system and observe how it reacts to make inferences about the underlying relationship between specific events and their effects. This is the strategy that underlies the most widely used effective connectivity technique, Dynamic Causal Modeling (DCM; Friston et al., 2003). Imaging data are collected across several related experimental conditions, and differential equations are used to model the changing relations between localized activation measures across conditions as a function of potential effective connectivity patterns. Bayesian techniques are then used to compare the model fit and identify models or classes of models that provide the best fit to the observations. This method is implemented in widely available statistical parametric mapping software and has been applied to both BOLD imaging and MEG/EEG data (Daunizeau et al., 2009; Valdes-Sosa et al., 2011). DCM has been continually refined since its development (Stephan et al., 2010). DCM has several vulnerabilities. The most critical is that it assumes that the same network, with the same underlying causal relations between elements supports processing across experimental conditions (Daunizeau et al., 2011). This assumption is at odds with evidence that flexible networks are assembled “on the fly” and evolve rapidly to address task and stimulus-specific processing challenges and opportunities (Buzsáki, 2006).

Recent statistical critiques further suggest that the potential model space is too large to allow exhaustive model evaluation (Lohmann et al., 2012). As a result, DCM is only practical given either very small networks examined across very few experimental conditions, or heavy reliance on *a priori* heuristic constraints on model space.

The method that we are presenting, Granger causality analysis relies on the temporal structure of causation. The germ of the idea, first proposed by Wiener (1956), and later realized more fully by Granger (1969) is that causes carry predictive information about an effect that is not present anywhere else in a system. Granger suggested that if you track everything and use that information to predict the future behavior of one variable, the most useful information will come from tracking the behavior of the variable that directly causes change in the variable you are trying to predict. Any quantitative prediction will be associated with an error term. Granger’s strategy was to make two predictions. The first prediction would be made based on measurements of all variables, including the one that you are trying to predict. The second prediction would be made based on a restricted set of measurements that would not include one variable. Granger argued that if the dropped variable directly influenced the predicted variable, the error term would become larger. Unlike DCM, this approach does not involve comparison across experimental conditions, and it does not involve the *a priori* specification of effective connectivity models.

The requirement that all potentially causal variables are tracked insulates Granger analysis against the effects of spurious correlation. In spurious correlation two variables that have no direct relationship appear to interact due to the influence of an unobserved mediating variable. For example, there may be a relationship between energy consumption and ice cream sales mediated by temperature. When it is hot people use more energy (for cooling) and may be more likely to buy ice cream (also for cooling). If this is the case, electricity use may predict ice cream sales, but temperature should be an even better predictor. One’s ability to forecast ice cream sales should not be reduced if they lose information about electricity use, as long as they retain information about temperature. However, assuming that energy use is also determined by many factors other than temperature, losing information about temperature should hurt the prediction of ice cream sales. Given the practical limitations on the measurement and analysis of brain activity, Granger analyses of neural data never fully meet this assumption. As a result, spurious correlation may occur. For this reason, researchers must make every effort to sample all potentially causal regions within the limits of their measurement techniques.

In some instances, Granger analysis is implemented using bivariate models. For example, given a three variable (A, B, C) system, they may break analyses down into separate models of the prediction of A by B, A by C, B by A, B by C, C by A, and C by B. This approach is mathematically straightforward, and is used widely in Granger analyses of imaging data (c.f. Hesse et al., 2003; Dhamala et al., 2008b). Despite its practicality, this approach is vulnerable to artifact from spurious correlation, even if all causal factors are sampled. Geweke (1982) addressed this problem by extending Granger analysis to the multivariate case in which all variables are entered into the prediction and a single variable is eliminated from the restricted model.

## Implementation

While the basic premise of causal inference in Granger analysis is simple, application of the technique involves a number of more technical considerations. These are explored in more depth in several reviews (Ding et al., 2006; Seth, 2010). The purpose of the current review is to identify constraints that have specific implications for imaging methodology or the form of cognitive models.

## Coverage, Spatiotemporal Resolution, and the Choice of an Imaging Modality

The ideal imaging modality for use with Granger analysis would: (1) independently monitor activity from all interacting brain regions, (2) provide sufficient spatial resolution to interpret the functional significance of measurements of local activity based on the existing BOLD imaging and neuropsychological localization literatures, and (3) provide sufficient temporal resolution to support reliable quantitative modeling and prediction of the time course of activation over the interval of interest. In this section we consider how current imaging modalities align with these requirements.

Constraints on temporal resolution all follow from the way predictions are made in Granger analysis. In its pure form, the logic of Granger analysis does not constrain the choice of how quantitative predictions are made. Discrete vector autoregression models are widely used (Geweke, 1982; Gow et al., 2008; Seth, 2010), but other techniques including time-varying Kalman filter based models (Havlicek et al., 2010; Milde et al., 2010) and non-linear models (Dhamala et al., 2008a) have also been used in neuroscience research. The choice of a modeling or prediction strategy is generally based on consideration of assumptions about the nature of underlying processes (e.g., whether they are adequately described by linear or non-linear models), ease of implementation, and technical considerations described below.

All of these methods are forms of time series analyses that depend on the availability of a critical mass of observations. Time series analyses based on too few observations are not considered reliable. The minimum number of observations depends on the specific choice of a modeling technique, the inherent variability of the data, and the number of parameters being modeled (Box et al., 1994). Spectral Granger analyses are further constrained by the choice of frequency range. Heuristic estimates about the minimum number of time samples for meaningful time series analysis may range between 50 and 200. When applied to long time intervals as in resting state analyses (Uddin et al., 2009) or the analysis of events that take place over relatively long time periods such as learning, habituation, or fatigue (Deshpande et al., 2009) the temporal resolution of BOLD imaging techniques is sufficient to meet this requirement. However, given current limitations on the temporal resolution of time-resolved fMRI it is unclear whether BOLD imaging data provide sufficient resolution to adequately model neural events that play out over tens to hundreds of milliseconds, such as those involved in the online comprehension and production of language. Roebroeck et al. (2005) and Stephan and Roebroeck (2012) suggest that BOLD data may be suitable for bivariate, but perhaps not multivariate, Granger analysis for these reasons.

Intracranial recording, EEG and MEG all offer millisecond resolution sufficient to perform meaningful modeling of event-related cognitive processing. However, each of these approaches present tradeoffs. Several studies have applied Granger analysis to intracranial electrode data (Brovelli et al., 2004; Dhamala et al., 2008a; Gow et al., 2009). Intracranial data provide extremely high spatial and temporal resolution. However, due to the invasive nature of this technique, it is only feasible to record over a very small area of cortex surrounding an area showing a suspected pathology in human subjects. This sparse sampling may be sufficient to analyze highly localized networks such as the cortical columns examined by Dhamala et al. (2008a) but is vulnerable to the effects of spurious correlation associated with unrecorded sources.

Magnetoencephalography and EEG present a different challenge. They provide temporal resolution sufficient to support modeling, but do not provide the localization accuracy associated with BOLD imaging or intracranial electrophysiological recording. One solution to this problem is to rely on sensor space analyses (Kaminski et al., 2001; Hesse et al., 2003). This sidesteps the localization problem, but limits the interpretability of the function of the brain activations that drive the analysis.

A number of techniques exist for localizing the source of measured MEG and EEG signals in the brain. For the purpose of Granger analysis it is necessary to use a method that can localize multiple distributed sources. Source localization is challenging because the inverse problem (inferring the location of sources based on measures made outside of the skull) is inherently ill posed. No set of measures projects to a unique source configuration. A number of techniques have been devised to address this problem including classical least squares minimum norm estimation or MNE (Hämäläinen and Ilmoniemi, 1984), its spatially normalized variant dynamic statistical parametric mapping or dSPM (Dale et al., 2000), and standardized low-resolution electromagnetic tomography or sLORETTA (Pascual-Marqui, 2002). Comparison of these techniques is not straightforward, because each offers different patterns of localization error, spatial extension (spatial spread of point sources), and amplitude estimation as a function of several factors including source location (Hauk et al., 2011). In our work, we rely almost exclusively on MNE. Within each of these approaches, source localization can be improved in several ways including depth weighting to correct a bias toward superficial sources (Lin et al., 2006b), using anatomical reconstructions based on MR data to constrain localization (Hämäläinen and Sarvas, 1989), the imposition of dipole orientation constraints (Lin et al., 2006a), and active correction for head movement artifacts (Wehner et al., 2008).

Source estimation is also improved by integrating multiple imaging modalities. A number of fusion strategies for combining BOLD and MEG or EEG data are currently in development or limited usage (Valdes-Sosa et al., 2009; Henson et al., 2010; McDonald et al., 2010; Lei et al., 2011; Luessi et al., 2011) and at least one study has applied Granger analyses to fused multimodal data (Lei et al., 2011). This type of data fusion is difficult due to the different temporal sensitivities of BOLD and MEG or EEG data. BOLD imaging priors may suggest a bias toward a source localization, but they typically do not provide information about when that source is active, and so may undermine localization of some of short-term activations.

Another approach is to integrate simultaneously collected MEG and EEG data. EEG and MEG signals are complementary signals arising from the same sources: synchronized post-synaptic currents produced in the vicinity of apical dendrites of pyramidal cells (Hämäläinen et al., 1993). The addition of EEG improves MEG localization in two ways. It increases the density of the senor array, and provides access to sources that MEG cannot detect because it because it is insensitive to radially oriented sources (Hämäläinen et al., 1993). In our work, we rely primarily on MRI-constrained MEG/EEG data (Gow et al., 2008; Gow and Segawa, 2009). These data have superior spatial resolution to either unimodal EEG or MEG, with typical localization errors in some regions on the order of 5–6 mm (Sharon et al., 2007).

While MRI-constrained combined MEG/EEG appears to offer the best currently available combination of spatial and temporal resolution and coverage, it has several limitations as a vehicle for Granger analysis. The most important is its inability to accurately track deep subcortical activations (Hämäläinen et al., 1993). As a result, some apparent cases of cortico-cortical interaction may be mediated by unmeasured subcortical structures.

## Stationarity

Classical Granger analysis assumes that all time series are stationary within the time period being modeled. This means that their means, variances, and covariances with other factors do not change over the period that is being analyzed. Granger (1969) argued that this assumption is necessary because causal relations may change within non-stationary time series, undermining modeling, and making it more difficult to define terms and assumptions in a manner that is verifiable. When Granger analysis is based on multiple vector autoregression (MVAR) models, non-stationary time series can produce an artifact called “spurious regression” in which correlations reflect non-stationarities instead of deterministic relations between variables (Granger and Newbold, 1974). The stationarity assumption creates significant challenges for the analysis of neural dynamics because they inherently evolve over time.

Stationarity can be evaluated in several ways. One approach is to generate a correlogram. A correlogram is a graph that plots the *k*th order autocorrelation coefficient as a function of *k*. In stationary time series autocorrelations quickly disappear as *k* increases (Box et al., 1994). A number of simple statistical tests including the augmented Dickey–Fuller test (Greene, 1997) can be used to verify stationarity by testing for the absence of unit roots. Unit roots are a characteristic of non-stationary processes that reflect a failure of values to regress to a common mean over time.

Time series can often be rendered stationary through transformation. The simplest approach is to difference (Seth, 2010). This involves using the first derivative of the time series rather than the raw values. Differenced time series are then tested for stationarity. If they are still non-stationary the process can be iterated until they pass the test. While computationally sound, this approach may obscure the interpretation of Granger results, which reflect velocity of acceleration of change in activation rather than raw activation itself. The other primary transformation strategy is to model the non-stationary component of time series and subtract that from the observed values. This can be done through simple linear detrending or may involve more sophisticated modeling techniques. In practice, differencing and detrending strategies may be applied in combination.

One way to reduce reliance on transformation is to conduct analyses over short windows during which time series may be locally stationary (Ding et al., 2000). In this strategy, analysis is conducted over a series of short overlapping windows, with MVAR parameters adapted as necessary at each successive window. This approach simplifies the interpretation of Granger results. While useful for bivariate analysis or multivariate analysis of very small networks, it becomes problematic for the analysis of larger models. The number of variables used in modeling grows as a function of the model order (the number of lagged units over which a prediction is made) and the number of time series that are used to predict a variable. When the number of variables approaches or exceeds the number of observations a model is overfit and thus unreliable. In our experience, this often makes it impossible to define an analysis window that is short enough to show local stationarity when realistically large sets of ROIs are used.

The stationarity assumption only holds if time series models apply to an interval of time. As a result, the stationarity assumption can be avoided if analyses are applied to a point in time rather than an interval. Several methods have been developed that take advantage of this fact by calculating instantaneous measures of Granger causation at each timepoint. One approach involves the use of a regressive least squares with forgetting algorithm to calculate autoregressive parameter matrices (Moller et al., 2001). More recently, researchers have turned to the use of Kalman filter techniques that may be used to model larger models (Milde et al., 2010). Kalman filter techniques have been applied to Granger analysis of BOLD, EEG, and multimodal data (Valdes-Sosa et al., 2009; Havlicek et al., 2010, 2011; Milde et al., 2010; Gow and Nied, 2012). Kalman filtering techniques appear to offer the best currently available means of both addressing the inherent non-stationarity of neural signals and meeting Granger analysis’ requirement of including all potentially causal signals.

## ROI Selection

The requirement to measure all potentially causal signals is both a fundamental strength of Granger analysis because it minimizes the likelihood of spurious correlation artifact, and a fundamental weakness because it is unrealistic. Granger (1969) described this assumption as being “completely unreal” (p. 429) and developed the application of his technique around the use of practically available measures.

In neuroscience applications the selection of regions of interest (ROIs) to track is limited by the choice of imaging techniques and the practical limits of computation. Intracranial recording techniques can only be applied to human subjects over a very limited area in the regions of suspected physiological abnormality in available presurgical patients. In BOLD and electrophysiological techniques the spatial resolution of analyses places constraints on the size and proximity of ROIs. Recognizing these constraints, analyses could in principle be applied to all measureable and non-redundant vertices or voxels. Depending on how strictly redundancy is defined, this could lead to the analysis of networks with hundreds to perhaps thousands of units. Given the computationally intensive nature of Granger analysis and bootstrapping methods for assessing the statistical significance of Granger causality measures, it would be impractical to run analyses involving so many variables.

Region of interest selection can be approached in several ways. One way is by performing analyses on sensor data using EEG or intracranial EEG (iEEG) data based on relatively few channels (Kaminski et al., 2001; Gow et al., 2009). For EEG data this approach comes with the cost of losing the ability to relate individual timeseries to what is known about the neural localization of function. In contrast, iEEG electrode data provide excellent implicit source localization, but as noted above, are impractical to deploy in a manner that allows sampling of all potentially causal signals except in the case of very localized discrete networks. Another approach is to select ROIs based on *a priori* theoretical considerations. This approach is at odds with the exploratory nature of Granger analysis. It also carries several risks. One is the scarcity of well-developed anatomically grounded models of cognitive task performance. Another is the fact that the models that do exist tend to involve a very small set of ROIs and may underestimate the size and extent of actual networks, opening the possibility of artifact by spurious correlation.

A more common approach is base ROI identification on simple measures of activation (Roebroeck et al., 2005; Sridharan et al., 2008). This method allows the inclusion of ROIs that are not predicted by theory. The primary limitation of this approach is that it may fail to identify ROIs that play a causal role in neural dynamics but are downregulated during task performance. Another problem is that this approach may fail to identify processing regions that show no overall increase or decrease in activation, but do show a qualitative change in the nature of activation such as a change in phase-locking that may influence activation in another region.

A principled approach is to base ROI selection on measures of functional connectivity such as correlated regional changes in BOLD activation, phase coherence, or phase-locking (Supp et al., 2007; Gow et al., 2008; Gow and Segawa, 2009). Gow et al. (2008) found that this approach identified a superset of ROIs identified by activation alone, including ROIs that did not show increased activation, but still exerted significant and theoretically important causal effects during processing.

This approach to ROI selection typically produces a larger set of ROIs than do hypothesis-driven effective connectivity studies. Recent studies of effective connectivity in language processing employing DCM have explored network models ranging in size from three to six nodes (c.f. Hara et al., 2007; Leff et al., 2008; David et al., 2011; Lee and Noppeney, 2011; Yvert et al., 2012). Given the hyperexponential growth in model space intrinsic to DCM, it is not computational reasonable to exhaustively test all potential models given networks of more than 3 nodes explored by more than 3 experimental conditions (Lohmann et al., 2012). As a result, DCM analysis can only be applied to very small models if the goal is to identify the best model. In contrast, Gow et al. (2008) identified nine ROIs in a study of lexical influences on phoneme categorization and Gow and Segawa (2009) identified 16 in a study of the perception of coarticulated speech. These higher numbers do not represent the upper limits on Granger analysis. Gow et al. (2009) applied the same analyses to a 64 electrode sensor space analysis of iEEG data using differencing to achieve stationarity. Given the concerns raised above about the interpretability of differenced data, we feel that Kalman filtering techniques that do not require the stationarity assumption are preferable. Milde et al. (2010) successfully applied this approach to the analysis of a 58 node network.

Regardless of how ROIs are initially identified, it is important that all timeseries associated with different ROIs are non-redundant. Granger analysis is premised on the role of unique predictive information. If two timeseries were too similar there would be no loss of information associated with a restricted model that eliminated one of those areas, even if one of those regions played a causal role in driving activation in the predicted region. As a result, the effect of including redundant timeseries should be reduced sensitivity. In MEG and EEG, redundancy may be arise due to spatial “smearing” or “leakage” intrinsic to source localization (Hämäläinen et al., 1993). Similarly, the low temporal resolution offered by BOLD imaging techniques my minimize differences between timeseries.

## Managing Output

The sheer volume of output produced by Granger analysis poses significant challenges for interpretation. Unlike hypothesis testing analyses in which the complexity of a possible result is constrained by the state of explicitly stated theory and the limits of experimental design, exploratory analyses such as Granger analysis may produce results that lie outside of current understanding or expectation. Data-driven ROI identification typically produces larger networks than those typically explored by techniques such as structural equation modeling. If one includes analyses of self-causation or Granger autonomy which are inherently one directional, the number of potential directed causal relationships between nodes is 2 × (number of ROIs)^2^ − number of ROIs. When measures of continuous Granger causation are applied these relationships may be fully evaluated at the frequency of the sampling rate. This means that analysis of a 20 ROI network employing a continuous Granger analysis of a MEG/EEG sampled at 1,000 Hz over 200 ms produces 156,000 comparisons, which may each be submitted to significance testing based on its own independent distribution.

This complexity may be approached in several ways. The simplest approach is to ignore most of the output and to focus on a small number of connections of interest. For example, when examining the role of lexical influences on speech perception, Gow et al. (2008) focused on the role of interactions between brain regions hypothesized to play a role in lexical representation and a brain region hypothesized to play a role in acoustic-phonetic processing. In order to ensure the integrity of the analysis, all nine ROIs were included in the analysis. The analyses were further simplified by the use of an epoch-based analysis focused on the interval in which STG activation diverged between experimental conditions. As a result, primary interpretation of the experiment rested on a single connection. Consideration of a handful of additional influences on STG and of the causal interactions that led to the initial activation of the lexical ROI provided further context for understanding this interaction.

**Figure 1:**
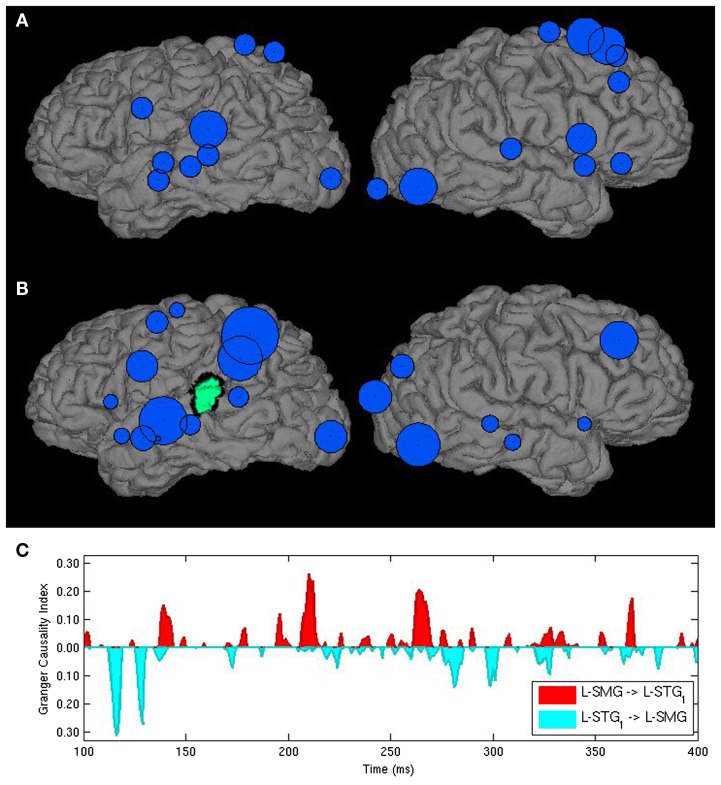
**Three Granger analyses for identifying critical processing interactions in a word-picture matching task**. **(A)** Is a bubble graph used to identify processing hubs. Bubbles are centered in ROI centroids and varying in size as a function of the number of ROIs providing significant Granger input over the analysis period. **(B)** Plots the relative strength of Granger influence (cumulative Granger Causality index values) by ROIs on a target region identified in green. **(C)** Plots a continuous measure of Granger causation strength for afferent (red) and efferent (teal) interactions between the left STG and SMG from 100 to 400 ms.

While this approach narrows the number connections to be considered, it may do so at the cost of ignoring other connections that may play a critical, but unexpected role in processing. For this reason, we developed another approach that relies on simple measures drawn from graph theory to guide purely exploratory analysis. The most basic of these is *degree*, which refers to the number of connections a node, or in this case, an ROI has with other components of the network. Degree can further be differentiated into *in-degree*, the number of nodes that have influence on a given node, and *out*-*degree*, the number of nodes that a given node influences. These measures can be weighted by connection strength as quantified by Granger index (Milde et al., 2010) or thresholded to include only those connections that are statistically significant or reach a particular threshold. Degree can be visualized by a source space bubble graph where the size of circles placed over ROIs depicts a degree measure either in static or movie form. ROIs that show high-degrees serve as processing hubs within a network (see Figure 1A). In particular, ROIs with high in-degrees may be interpreted as regions where multiple information types are integrated. Similarly, a large out-degree identifies a node that drives multiple operations. Having identified computationally critical nodes based on these measures, researchers may focus on the interpretation of the subsets of connections that contribute to those degree measures (Figure 1B), examining time-varying graphs of continuous Granger index measures for a specific directed connection or set of connections as a final stage to understand how microprocessing interactions are organized in time (Figure 1C).

This strategy also has its vulnerabilities. The most critical is that it may over-represent the role of processing hubs in the overall dynamics that support cognitive processes. For this reason, it may be advisable to take a hybrid approach that combines question or hypothesis-driven interpretation with additional exploratory analyses guided by graph theory.

## Summary of Tutorial

The preceding tutorial provides the outline of an at-present-unique approach to Granger analysis premised on the importance of respecting the assumptions that underlie the logic of Wiener and Granger’s method of causal inference. We are advocating an approach to Granger analysis that involves the use of: (1) high spatiotemporal resolution imaging techniques capable of sampling all brain regions, (2) functional connectivity analyses to identify the set of all brain regions that interact during processing, (3) time-varying VAR modeling techniques to provide full multivariate modeling and prediction involving a realistically large set of ROIs, and (4) graph theoretic techniques to identify the loci of fundamental processing interactions. These properties, coupled with Granger analysis’s ability to identify directed causal interactions without the use of an *a priori* processing model, produce results that show linguistic and cognitive processes in a different light. In the remainder of this paper we will review some of our own work and explore the implications of this approach for understanding linguistic processes and their biological bases.

## Granger Analysis Studies of Speech Processing

Work in our lab has focused on the use of these methods to examine the neural dynamics that support spoken language processing. In this section we will briefly describe two representative studies that illustrate the application of this approach. Both studies relied on the same processing stream. Subjects performed a behavioral task while simultaneous EEG and MEG data were acquired. These were reconstructed with MRI anatomical constraints as source space MNE activation movies. ROIs were identified in these movies based on 40 Hz phase-locking to a reference region identified in the left STG that showed early and persistent activation during task performance. We compared the timing of major activation maxima and minima for all pairs of timeseries to eliminate redundant timeseries. Discrete VAR Granger analyses were then performed on these timeseries data using the Seth’s (2010) MATLAB Granger Analysis Toolbox. Stationarity was achieved by differencing.

Gow et al. (2008) examined the role of top-down lexical influences on speech perception. In this study subjects heard tokens from *a* /*s*/-/∫/ continuum in the context of lexical contexts that biased interpretation toward either /s/ (*_andal*) or /∫/ (*_ampoo*) and performed an explicit fricative categorization task. Behavioral analyses replicated the classical Ganong (1980) effect with listeners interpreting the same ambiguous fricative as /s/ in the *_andal* context and /∫/ in the *_ampoo* context. This behavioral effect is consistent with two contrasting analyses. One analysis suggests that lexical factors influence perceptual processing. The other is that lexical factors have no influence on perceptual processing, but may interact with the output of perceptual processing at a late decision stage (Norris et al., 2000). An fMRI study by Myers and Blumstein (2008) implicated increased bilateral posterior superior temporal gyrus (pSTG) activation in this process but could not discriminate between top-down and bottom-up accounts. The Gow et al. study found similar patterns of activation using MRI-constrained MEG/EEG, but identified a larger network based on an analysis of 40 Hz phase-locking to a reference area in the left pSTG. Granger analysis of this network revealed a complex pattern of causality characterized by early activation driven by left pSTG (from 80 to 280 ms post stimulus onset) followed by a period coincident with lexical access (280–480 ms) in which left pSTG activation was influenced by five different brain regions including the left supramarginal gyrus (SMG), a region implicated in lexical representation (Gow, 2012). To our knowledge, these were the first data that uniquely indicated a role of top-down influences on speech processing.

Gow and Segawa (2009) used the same imaging and analysis techniques to study the mechanisms that allow listeners to compensate for assimilation in the perception of spoken words. This effect has been broadly studied, and has been variously attributed to inference based on abstract phonological knowledge (Gaskell and Marslen-Wilson, 1996), the perceptual grouping of phonetic feature cues (Gow, 2003), or perceptual processes mediated by articulatory representation (c.f. Liberman and Mattingly, 1985). The last account, articulatory mediation, has been the target of intense investigation over the last 50 years, but has been difficult to evaluate due to the challenges of independently manipulating speech articulation and acoustic structure and determining whether articulation-related activation in perceptual tasks influences perceptual analysis.

In this work subjects performed a delayed sentence-picture matching task with stimuli in which spontaneously produced assimilation produced words that were phonetically ambiguous (e.g., *pen* pronounced $pe_{m}^{n}$). Analyses focused on the first 200 ms following the onset of word that immediately followed this item. Granger analysis of trials in which listeners showed behavioral evidence of compensation for items in which assimilation did not produce a potential lexical competitor to the intended word (e.g., $pe_{m}^{n}$) showed influence by both the left posterior middle temporal gyrus (pMTG) a region implicated in lexical representation (Gow, 2012), and the left dorsal premotor area, a region involved in articulatory representation (Watkins et al., 2003; Hickok and Poeppel, 2004) on the left pSTG. The same patterns were found in trials where assimilation produced a potential lexical competitor ($gu_{m}^{n}$) in the context of a much more complicated pattern of interactions that included confluent influences of 15 brain regions including many right hemisphere and non-perisylvian regions that are not typically associated with speech perception. These included regions associated with decision-making (left anterior and posterior cingulate), vision (superior occipital sulcus, primary visual cortex), and memory (left parahippocampal region). These results affirm a causal role of articulatory mediation in speech perception, and suggest that stimulus and task-specific factors play a major role in determining how the brain processes speech.

## Implications

### Interactive processing

The hypotheses that these studies address about the role of articulatory and lexical mediation in speech processing are not new. In each case we have used Granger analysis to examine hypotheses that have resisted verification or falsification due to the inherent limits of behavioral and BOLD imaging research paradigms. Different experimental and analytical techniques often support different types of inferences. A primary limitation of behavioral data, simple BOLD subtraction, and even measures of functional connectivity is that they do not support strong inferences about the role of top-down mechanisms in processing. Abstractly, negative feedback produces self-limiting processes, while positive feedback accelerates processes and can move systems toward discrete states. The central role of feedback in domains as diverse as chemistry, electrical engineering, and systems biology is beyond question.

For all of its computational merits, there are serious reasons to doubt both the necessity of top-down processes and the inference that evidence of interaction between higher and lower level representations indicates the existence of top-down processes. Dennett (1992) makes the distinction between bottom-up processes that “figure out” (e.g., identify an ambiguous speech sound to recognize a word), and top-down processes that “fill in” (e.g., modify lower level phonetic representations to conform to the lexical hypothesis). The argument is that if the purpose of the process is to arrive at the correct output representation, there is simply no need to change the input representation. Norris et al. (2000) developed this argument further, demonstrating that the purely feedforward MERGE model could account for the interaction between phonetic and lexical knowledge in a variety of behavioral results given the assumption that phonetic representation feeds lexical processes, and that the output of both lexical and phonetic processes are combined at a later decision process without feedback. This structural interpretation could be applied to evidence of articulatory mediation in speech perception or any data that show the combined influence of multiple types of information on an output measure.

This is a critical distinction, but one that cannot be explored purely behaviorally, because behavioral methods only directly tap a decision, and (with the exception of mouse- and eyetracking) only do so at one point in time, typically after the processing dynamic is complete. Measures of localized neural activation have the advantage of offering the ability to observe multiple localized processors. Here, top-down lexical processes may produce increased activation of acoustic-phonetic processors (Myers and Blumstein, 2008). However, top-down processes are generally associated with ambiguous input, and so this increased activation could be attributed either to top-down facilitation or the need for increased bottom-up processing to address the ambiguity. Correlational measures of functional connectivity also fall short because both feedforward and feedback mechanisms can produce correlated activity between two linked processors. Our Granger causality results thus provide unique evidence for top-down influences on perceptual processing.

### Functional architecture: Streams versus hubs

The most surprising and consistent result that we have found in our speech studies is evidence that activation in bilateral pSTG is directly influenced by a large bilateral network. This finding is at odds with the metaphor of the processing stream that has dominated cognitive psychology, psycholinguistics, and current neuroanatomical models of language processing (c.f. Denes and Pinson, 1993; Scott and Wise, 2004; Hickok and Poeppel, 2007; Rauschecker and Scott, 2009; Sahin et al., 2009). The stream metaphor suggests that processing occurs through a sequence of ordered steps in multiple parallel processing streams. In its most concrete form, the stream metaphor suggests a linear cascade of ordered processes in which the output of one process provides the input to the next. Within the dual stream model of language processing (Hickok and Poeppel, 2007), the pSTG has bidirectional connectivity with the mid-posterior superior temporal sulcus, which becomes the origin for both the ventral and dorsal processing streams. However, even advocates of interactive processing may recognize the general outline of a stream in models such as TRACE (McClelland and Elman, 1986) in which bidirectional connectivity is limited to two adjacent levels within a network.

Our results suggest that bilateral pSTG serves as a processing hub where information from a large, bilateral network of traditionally recognized perisylvian language regions as well as regions associated with non-linguistic, but task relevant representation and processing are integrated. In network science, the term hub refers to a high-degree node (a node that is connected to many other nodes). Recent studies of white matter tractography and functional connectivity present converging evidence that hub organization is a fundamental property of brain structure (c.f. Bassett and Bullmore, 2006; Bullmore and Sporns, 2009). Using a combination of anatomical and functional connectivity analyses, Hagmann et al. (2008) identified bilateral superior temporal cortex as a major hub based on six different connectivity measures. This role is not unique within language processing. Related anatomical and functional analyses have identified other likely language processing hubs including the middle temporal gyrus, temporal poles, SMG, and inferior frontal lobes (c.f. Patterson et al., 2007; Hagmann et al., 2008; Tomasi and Volkow, 2010).

Hub architecture has several advantages. In the context of a “small world” organization characterized by a high-degree of local connectivity combined with a distributed pattern of hubs showing longer range connectivity, hubs help reduce the number of links needed to connect any two points in the cortex (Bassett and Bullmore, 2006). This may contribute to flexibility in processing by allowing direct or almost direct communication between almost any pair of regions in the brain. Evidence that hubs tend to show high connectivity with other hubs, a pattern known as “rich-club” organization (Van Den Heuvel and Sporns, 2011), further supports integration over a distributed network and provides a physical basis for interactions in which many types of representations and analyses can mutually constrain and support one another.

The role of a hub is to integrate. In the case of pSTG, hub status provides a ready mechanism for explaining a large body of behavioral evidence that suggest that the perception of speech is influenced by diverse factors including coarticulation (Liberman, 1957), phonotactic context (Massaro and Cohen, 1983), lexicality (Ganong, 1980), semantic context (Warren and Warren, 1970), syntactic and prosodic context (Price and Levitt, 1983), and previous exposure to a dialect or speaking style (Kraljic et al., 2008; Maye et al., 2008). The ability to flexibly draw on many types of representations contributes to the robustness of speech perception. Moreover, to the degree that pSTG connectivity is reciprocal, hub status also serves a parity checking function, assuring that all levels of representation are drawing on a common interpretation of speech sounds. A broader pattern of rich-club organization may amplify these functional properties by allowing many levels of linguistic representation to reinforce and support one another.

### Interpreting function

The goal of our effective connectivity analyses of source space activation is to identify how specific types of information interact to achieve a processing goal. The validity of these analyses rests in large part on how accurately we are able to ascribe functional significance to activation in any particular brain region. For example, claims that lexical representations influence speech processing in the examples above depend on the reliable inferences about the functional significance of activation in pSTG, SMG, and MTG (Gow et al., 2008; Gow and Segawa, 2009). In this section, we will examine how conversely, analyses of effective connectivity may inform our understanding of functional localization.

The functional localization literature relies primarily on pathonormal inference and BOLD imaging data. The inferential limits of both techniques have been well explored. Neuropsychologists depend on the assumption that damage to localized processors does not influence how intact portions of a processing network operate (Kosslyn and Intrilligator, 1992). Similarly, the interpretation of BOLD imaging data by the subtraction technique depends on the assumption that there are no interactions between processors (Friston et al., 1996). Both of these assumptions are belied by the rich, dynamic pattern of interaction between brain regions seen in studies of effective connectivity. Friston et al. recognized this problem and suggested that a complete accounting of functional localization requires the characterization of both local activation and patterns of interaction.

Consider again the role of pSTG in the examples described above. Bilateral pSTG appears to play a role in higher-level spectrotemporal analysis of sound. This interpretation is consistent with pSTG’s proximity to primary auditory cortex and a pattern of reliable activation in subtractions between listening to complex auditory stimuli and rest (Hickok and Poeppel, 2007). More focused analyses suggest that this region shows some category-specific sensitivity to acoustic-phonetic cues (Blumstein et al., 2005). This simple view of bilateral pSTG appears to be at odds though a variety of results showing pSTG activation in both speech production and perception tasks (Buschsbaum et al., 2001) and sensitivity to manipulations of speech and non-speech factors including lexicality (Gow et al., 2008), morphology (Tyler et al., 2005), grammaticality (Raettig et al., 2010), the producibility of non-native speech sounds (Wilson and Iacoboni, 2006), spatial congruity between sound sources and semantically related targets (Plank et al., 2011), unaltered versus scrambled environmental sounds (Thierry et al., 2003), and the presence of melodic pattern in a tone sequence (Griffiths et al., 1998).

This diversity of effects may reflect a kind of referred sensitivity, in which BOLD sensitivity to a stimulus property such as morphology or melodic structure in pSTG may not indicate that it is directly involved in the detection or representation of that process. Instead, it may reflect influence of another localized processor that is sensitive to those properties on the activation of pSTG. This observation has bearing on the historic debate over where the analysis of speech sounds diverges from the analysis of non-speech sounds. In this framework, the same region may play a role in general auditory processing for many sounds, but play a role in language-specific processing when it becomes involved in reciprocal processing interactions with a brain region involved in specifically linguistic representation.

The use of temporally continuous measures of Granger causation (Milde et al., 2010) or measures of Granger causation over multiple discrete time windows (Gow et al., 2008) further demonstrate that processing interactions evolve over time. This may be attributable to differences in the rise time associated with different linguistic processes. For example, Gow et al. (2008) found increased pSTG activation within 100 ms of the onset of spoken words, but found no effect of lexicality on pSTG activation until 220 ms after the word onset. This suggests that pSTG activation reflected only non-lexical auditory processes until listeners had heard enough of words to identify lexical candidates. This result parallels the finding by Sahin et al. (2009) that Broca’s area is sensitive to different types of properties at different points in time. In our experiment, top-down lexical influences produced a behavioral trend toward normalization, with the categorization of ambiguous speech sounds shifting to support lexical candidate. This finding was confirmed in later experiments examining the role of lexical mediation in phonotactic influences on speech perception (Gow and Nied, 2012). This evolution of influences is consistent with the possibility that pSTG activation initially reflects raw auditory stimulus properties, but is normalized through resonant processing interactions with stored lexical representations to approximate acoustic-phonetic and later abstract phonological representations of speech. This hypothesis requires further evaluation, but it illustrates the notion, raised by high spatiotemporal resolution analyses of causal brain interactions, that functional localization analyses need to identify a region’s function in the context of a network, determined by a task, and localize that function in both time and space.

## Summary

Granger analysis of high spatiotemporal resolution data such as MRI-constrained MEG/EEG provides a powerful tool for analyzing directed causal interactions between brain regions during event-related processing. These analyses are rooted in non-technical intuitions that human observers use to identify patterns of everyday causation. The logic of these inferences constrains methodological choices about suitable imaging modalities. Statistical considerations further constrain the implementation of these analyses and favor the use of methods that provide continuous measures of causality based on full multivariate modeling. We have suggested that the requirements of these analyses produce results that point to unexpected levels of complexity and interaction in language processing and inform the way that we understand functional localization and characterize the functional architecture that supports language processing.

## Conflict of Interest Statement

The authors declare that the research was conducted in the absence of any commercial or financial relationships that could be construed as a potential conflict of interest.
